# Self-Objectification and Adolescent Depressive Symptoms: A Three-Wave Moderated Indirect-Effects Model Involving Body Shame and Self-Disgust

**DOI:** 10.3390/bs16071153

**Published:** 2026-07-09

**Authors:** Liming Yue, Yang Cui, Xiangping Gao

**Affiliations:** Department of Psychology, Shanghai Normal University, Shanghai 200234, China; 1000495286@smail.shnu.edu.cn (L.Y.); 1000529945@smail.shnu.edu.cn (Y.C.)

**Keywords:** self-objectification, body shame, self-disgust, depression, longitudinal study

## Abstract

Background: Self-objectification has been consistently associated with adolescent depressive symptoms; however, the psychological processes linking appearance-based self-evaluation with broader self-directed negativity remain unclear. Objective: This three-wave study examined whether body shame and self-disgust were involved in the longitudinal association between self-objectification and depressive symptoms. It also tested perceived peer connectedness, indexed by reverse-coded loneliness, as a contextual moderator and used network analysis to explore item-level associations between body shame and self-disgust. Methods: A total of 1181 Chinese adolescents completed measures of self-objectification and perceived peer connectedness at Time 1, body shame and self-disgust at Time 2, and depressive symptoms at Time 3. Path analysis within a structural equation modeling framework tested a moderated indirect-effects model controlling for gender and grade. Results: Self-objectification was longitudinally associated with depressive symptoms both directly and indirectly through body shame and self-disgust. Perceived peer connectedness modestly attenuated the association between self-objectification and body shame. Network analysis identified item-level bridge associations between body shame and self-disgust. Conclusions: The findings suggest that self-objectification may be linked to later depressive symptoms through appearance-specific shame and broader self-directed negativity. Perceived peer connectedness showed a modest buffering pattern, and the network results highlighted item-level links between body shame and self-disgust that may inform future research on adolescent body-related self-evaluation.

## 1. Introduction

### 1.1. Body Shame and Self-Disgust: From Appearance-Specific Evaluation to Broader Self-Directed Negativity

During adolescence—a critical period of identity formation—physical appearance becomes central to self-concept construction (Rodgers & Melioli, 2016). Negative body image has been consistently associated with increased depressive symptoms, reduced well-being, and elevated risk for disordered eating and related psychological difficulties (Zhao et al., 2024). To explain these associations, Objectification Theory (Fredrickson & Roberts, 1997) proposes that in appearance-focused sociocultural contexts, individuals internalize an observer’s perspective on their bodies and adopt external standards as a basis for self-worth (McKinley & Hyde, 1996). When perceived appearance falls short of these internalized ideals, individuals may experience body shame, characterized by self-blame and feelings of inadequacy. Empirical research has shown that self-objectification is associated with body shame, which in turn is linked to depressive symptoms and related psychological difficulties (Grabe et al., 2007; Szymanski & Henning, 2007; Saunders et al., 2024; Lang & Guo, 2025). Contemporary appearance-based environments may further intensify these processes during adolescence. Social media platforms expose adolescents to idealized and often edited appearance images, appearance-based feedback, and upward appearance comparisons, all of which may strengthen the internalization of appearance ideals and heighten body-related self-evaluation (Choukas-Bradley et al., 2022; Nerini et al., 2024; Tiggemann & Slater, 2015). Recent developmental–sociocultural perspectives suggest that adolescent body image concerns should be understood within broader digital and peer evaluative contexts, where appearance becomes a salient basis for social comparison and self-worth (Di Gesto et al., 2025).

However, prior studies have largely conceptualized body shame as a discrete appearance-related emotional outcome and have paid limited attention to whether it is connected with broader forms of self-directed negativity during adolescence. From a developmental perspective, body shame may be closely associated with broader negative self-evaluations, such as self-disgust. To clarify this conceptual link, it is important to distinguish body shame from self-disgust. Body shame refers to a negative self-conscious emotion centered on the body and appearance, particularly feelings of shame, inadequacy, and self-blame when one perceives the body as failing to meet internalized appearance standards. In contrast, self-disgust refers to a broader form of self-directed aversion and rejection, involving negative evaluation of the self as a whole rather than dissatisfaction with specific bodily attributes (Overton et al., 2008; Spreckelsen et al., 2018). Thus, although body shame and self-disgust are conceptually related, they differ in their evaluative scope: body shame is primarily appearance-specific, whereas self-disgust reflects a more global negative orientation toward the self. During adolescence, when self-concept remains developing and sensitivity to social evaluation is heightened (Guo & Wu, 2024), appearance-based evaluations may become closely linked to broader self-evaluative processes. Self-Discrepancy Theory suggests that perceived discrepancies between the actual and ideal self evoke shame and dejection, emotions closely linked to depressive outcomes (Higgins, 1987). In the domain of body image, repeated experiences of unmet appearance standards may gradually foster a sense of helplessness and stable, self-defining negative attributions (Stice et al., 2011; Blundell et al., 2024). Compared with body shame, self-disgust reflects a more pervasive and enduring rejection of the self as a whole (Overton et al., 2008; Spreckelsen et al., 2018) and is strongly associated with depression (Gao et al., 2022). From a developmental perspective, self-objectification may therefore be longitudinally associated with depressive symptoms through a theoretically specified indirect pathway involving body shame and self-disgust (Saunders et al., 2024). In the present study, cognitive expansion is used to describe the theoretically proposed broadening from appearance-specific negative evaluation to broader negative self-evaluation, grounded in self-discrepancy theory and adolescent self-concept development.

Building on this literature, the present study extends prior mediation models that primarily focused on body shame as an appearance-specific emotional consequence by examining whether self-disgust, a broader form of self-directed negativity, is involved in the longitudinal association between self-objectification and depressive symptoms. This integration allows the model to connect objectification theory with broader self-evaluative and affective processes in adolescence.

### 1.2. Perceived Peer Connectedness as a Small Contextual Buffer

Body shame and self-disgust may be closely connected during adolescence, but the strength of body-related self-evaluation may vary across social contexts. From a developmental contextual perspective, perceived peer connectedness may serve as a contextual moderator. During adolescence, social orientation shifts increasingly toward peers, who function as central sources of emotional support, social comparison, and self-evaluation (Brown & Larson, 2009; Choukas-Bradley et al., 2022). Consistent with social comparison theory, adolescents rely heavily on peer feedback to calibrate their self-worth, and appearance-focused peer contexts may make physical attractiveness a salient basis for self-evaluation (Festinger, 1954; D. C. Jones et al., 2004). Peer groups may also reinforce appearance-based norms through appearance-related comments, teasing, exclusion, or approval, thereby strengthening the association between self-objectification and body shame (D. C. Jones et al., 2004; Wang et al., 2019). In contrast, supportive friendships may provide emotional validation and affirmation in domains beyond appearance, such as personality, competence, and social belonging, which may reduce the centrality of physical attractiveness in adolescents’ self-evaluative systems (Bukowski et al., 2010; Furman & Rose, 2015; Lawler & Nixon, 2011). In such supportive contexts, appearance-based shame may be attenuated rather than generalized. Taken together, perceived peer connectedness may moderate the association between self-objectification and body shame, such that this association is weaker among adolescents reporting higher perceived peer connectedness.

### 1.3. Network Analysis: Exploring Item-Level Associations Between Body Shame and Self-Disgust

Although structural equation modeling (SEM) allows the examination of longitudinal associations among self-objectification, body shame, self-disgust, and depression at the macro level, it typically treats latent constructs as internally homogeneous. Consequently, SEM cannot identify which specific experiential components within body shame are most strongly connected with self-disgust at the item level. Network analysis provides a complementary framework to address this limitation. Rather than viewing psychological constructs as reflections of a single latent trait, the network approach conceptualizes them as systems of interacting symptoms or experiences (Epskamp et al., 2018). Within such models, bridge nodes linking distinct item communities may indicate potential points of connection between psychological domains (P. J. Jones et al., 2021). This approach is particularly relevant here because body shame and self-disgust are conceptually related but differ in evaluative scope: body shame is primarily appearance-specific, whereas self-disgust reflects a broader negative orientation toward the self. In the context of body image, certain body shame experiences may be more strongly associated with self-disgust and may therefore represent statistically central item-level links between the two constructs. By estimating bridge expected influence, the present study identifies the item-level components that connect the body shame and self-disgust communities, thereby providing exploratory item-level information about their association. Integrating longitudinal path analysis with network modeling therefore allows the present study to examine both longitudinal associations among constructs and exploratory item-level associations between body shame and self-disgust.

### 1.4. The Present Study and Hypotheses

Building upon the preceding theoretical integration, the present study employed a three-wave longitudinal design to construct a comprehensive model incorporating both theoretically specified indirect associations and contextual moderation. The aim was to systematically examine the psychological processes involved in the longitudinal association between self-objectification and depressive symptoms. Specifically, we propose that self-objectification is expected to be indirectly associated with depressive symptoms through body shame and self-disgust. In addition, perceived peer connectedness is introduced as a key contextual variable to test its moderating role in the pathway from self-objectification to body shame.

Accordingly, the following hypotheses were proposed:

**H1.**  *Self-objectification is expected to be positively associated with later body shame, self-disgust, and depressive symptoms*.

**H2.**  *Body shame and self-disgust are expected to show significant indirect associations linking self-objectification to depressive symptoms*.

**H3.**  *Self-objectification is expected to be indirectly associated with depressive symptoms through a theoretically specified pathway involving body shame and self-disgust*.

**H4.**  *Perceived peer connectedness is expected to moderate the association between self-objectification and body shame, such that the positive association is weaker at higher levels of perceived peer connectedness*.

**H5.**  *Perceived peer connectedness is expected to moderate the strength of the theoretically specified indirect association*.

Furthermore, to address the limitations of macro-level path models in capturing within-construct heterogeneity, the present study incorporates network analysis to explore the micro-level associations between body shame and self-disgust at the item level. We expect to identify key bridge nodes that connect the two symptom communities, thereby providing fine-grained information about item-level links between body shame and self-disgust and generating hypotheses for future intervention research.

## 2. Methods

### 2.1. Participants

Participants were recruited from one large public secondary school in southwestern China. The school was selected by convenience because of administrative feasibility and cooperation with the research team. Within the school, cluster sampling was conducted at the class level. Specifically, 31 classes were included, consisting of 15 Grade 7 classes and 16 Grade 10 classes, and all students in the selected classes were invited to participate. Thus, the sampling procedure should be understood as class-level cluster sampling within a single conveniently selected school. At Time 1, 1245 adolescents (52.0% girls; *M*age = 15.0, *SD* = 1.7; range = 12–18) participated. To facilitate longitudinal follow-up, students were primarily from Grades 7 and 10. Across three waves over one academic year, 1181 adolescents completed all assessments (retention rate = 94.8%). After excluding invalid responses and excessive missing data, the final analytic sample included 1181 participants. Independent-samples *t* tests indicated no significant differences between retained and attrited participants on demographic or core study variables (*p*s > 0.05).

### 2.2. Procedures

The study employed a three-wave longitudinal design over one academic year, with approximately six months between adjacent waves. This interval was chosen because one academic semester represents a meaningful developmental period in school-based adolescent research and allows sufficient time for appearance-related self-evaluation and emotional adjustment to unfold. At Time 1, which occurred at the beginning of the fall semester, demographics, self-objectification, and perceived peer connectedness were assessed. At Time 2, approximately six months later, body shame and self-disgust were assessed. At Time 3, approximately six months thereafter, depressive symptoms were assessed. Thus, the measurement schedule followed the theoretically specified temporal ordering of the model. Data were collected in school computer laboratories during scheduled classes and administered by trained graduate assistants. The study was approved by the university ethics committee. Written informed consent was obtained from education authorities, school administrators, parents/guardians, and all participants.

### 2.3. Measures

All measures were administered in Chinese. Published Chinese versions or Chinese versions previously used in adolescent or youth samples were adopted for all focal constructs. For scales originally developed in English, we used the available Chinese versions and reviewed the item wording to ensure that the items were age-appropriate and understandable for secondary school students. Internal consistency coefficients for all scales in the present sample are reported below.

#### 2.3.1. Self-Objectification

Self-objectification was assessed using the body surveillance subscale of the Objectified Body Consciousness Scale (OBCS; McKinley & Hyde, 1996). This subscale consists of eight items and measures habitual monitoring of one’s physical appearance and concern about how the body is viewed by others. A sample item is “I often worry about how my body looks to other people.” Items were rated on a 7-point Likert scale ranging from 1 (strongly disagree) to 7 (strongly agree), with higher total scores indicating higher levels of self-objectification. The Chinese version of the OBCS has been used in Chinese samples and has demonstrated acceptable psychometric properties (Chen & Jiang, 2007). In the present study, the body surveillance subscale demonstrated good internal consistency (Cronbach’s α = 0.86).

#### 2.3.2. Body Shame

Body shame was measured using the body shame subscale of the OBCS (McKinley & Hyde, 1996). This subscale includes eight items assessing shame, self-blame, and feelings of inadequacy when one perceives the body as failing to meet internalized appearance standards. A sample item is “When I feel that my body does not meet societal standards of appearance, I feel ashamed.” Participants rated each item on a 7-point Likert scale ranging from 1 (strongly disagree) to 7 (strongly agree), with higher total scores reflecting greater body shame. The Chinese version of the OBCS has been used in prior research with Chinese samples and has demonstrated acceptable psychometric properties (Chen & Jiang, 2007). In the present study, the body shame subscale showed good internal consistency (Cronbach’s α = 0.89).

#### 2.3.3. Self-Disgust

The Self-disgust was assessed using the Self-Disgust Scale (SDS; Overton et al., 2008). The scale consists of 12 items designed to measure feelings of aversion, repulsion, and rejection directed toward the self. A sample item is “I find myself disgusting.” Participants rated each item according to their experiences over the past six months on a 7-point Likert scale ranging from 1 (strongly disagree) to 7 (strongly agree). Total scores were computed, with higher scores indicating greater self-disgust. The Chinese version of the SDS has demonstrated good reliability and validity in Chinese samples (Xiong et al., 2019). In the present study, the scale exhibited excellent internal consistency (Cronbach’s α = 0.91).

#### 2.3.4. Depressive Symptoms

Depressive symptoms were assessed using the 10-item Center for Epidemiologic Studies Depression Scale (CESD-10; Andresen et al., 1994). The CESD-10 measures the frequency of depressive symptoms during the past week. A sample item is “I felt depressed.” Items were rated on a 4-point Likert scale ranging from 0 (rarely or none of the time) to 3 (most or all of the time). Total scores were computed by summing responses across items, with higher scores indicating more severe depressive symptoms. The CESD-10 has demonstrated good reliability and validity in Chinese adolescent and adult populations (Cheng & Chan, 2005). In the present study, CESD-10 scores were analyzed as continuous scores; cutoff scores were not used because the aim was to examine dimensional associations rather than to classify probable depression. Internal consistency in the present study was good (Cronbach’s α = 0.83).

#### 2.3.5. Perceived Peer Connectedness

Perceived peer connectedness was indexed using reverse-coded scores from the 6-item UCLA Loneliness Scale (ULS-6; Xu et al., 2018). The ULS-6 assesses perceived loneliness and social disconnection in interpersonal contexts. A sample item is “I feel left out.” Items were rated on a 4-point Likert scale ranging from 1 (never) to 4 (often). Scores were reverse-coded and summed so that higher scores indicated lower loneliness and greater perceived peer connectedness. The ULS-6 has shown good reliability and validity in Chinese samples (Xu et al., 2018). In the present study, the scale demonstrated good internal consistency (Cronbach’s α = 0.88).

#### 2.3.6. Data Analytic Strategy

All statistical analyses were conducted using SPSS (Version 28.0), Mplus (Version 8.4), and R (Version 4.4.2). Descriptive statistics and Pearson correlation coefficients were first computed for all study variables using SPSS. Gender and grade were included as covariates in all subsequent analyses, given their potential associations with self-perception and psychological adjustment during adolescence. Missing data were handled using full information maximum likelihood (FIML) estimation under the assumption of missing at random. FIML incorporates all available data into parameter estimation without imputing missing values and has been shown to yield unbiased parameter estimates and standard errors in structural equation modeling. This approach was implemented in all structural equation models estimated in Mplus. The SEM model followed the planned measurement schedule described above.

#### 2.3.7. Path Analysis and Moderated Indirect-Effects Model

Observed-variable path analysis was conducted in Mplus 8.4 using robust maximum likelihood estimation (MLR) to test the hypothesized indirect-effects model. All focal constructs were represented by observed composite scale scores rather than latent variables. Composite scores were used because the primary aim was to examine theoretically specified indirect associations and moderation effects, and all scales showed acceptable to excellent internal consistency in the present sample. Because the primary model used observed composite scores, separate latent measurement models were not estimated before the structural model. Autoregressive paths were not included because the focal constructs were measured at specific waves according to the planned temporal model rather than repeatedly assessed at each wave. Self-objectification was specified as the predictor, body shame and self-disgust as theoretically ordered mediators, and depressive symptoms as the outcome. A direct path from self-objectification to depressive symptoms and specific indirect paths through body shame and self-disgust were included.

Model fit was evaluated using the comparative fit index (CFI), Tucker–Lewis index (TLI), root mean square error of approximation (RMSEA), and standardized root mean square residual (SRMR). Following established recommendations (Hu & Bentler, 1999), values of 0.90 or higher for CFI and TLI and 0.08 or lower for RMSEA and SRMR indicated acceptable fit.

To test moderated indirect effects, perceived peer connectedness was specified as a moderator of the association between self-objectification and body shame. Continuous predictors were mean-centered, and an interaction term was included. Indirect effects and the index of moderated mediation were estimated using 5000 bias-corrected bootstrap samples with 95% confidence intervals (Preacher & Hayes, 2008; Hayes, 2015). Effects were considered significant when confidence intervals did not include zero. Significant interactions were probed using simple slope analyses at low, mean, and high levels of perceived peer connectedness.

To address measurement comparability across gender, supplementary multi-group confirmatory factor analyses were conducted to examine configural, metric, and scalar invariance for the focal scales, consistent with the importance of evaluating cross-group measurement comparability in adolescent research (Baroni et al., 2023).

#### 2.3.8. Network Analysis

To further examine item-level associations between body shame and self-disgust, network analysis was conducted using Wave 2 data in R (Version 4.4.2) with the bootnet, qgraph, and networktools packages. A Gaussian graphical model (GGM) was estimated, in which nodes represented the 8 body shame items and the 12 self-disgust items, and edges reflected regularized partial correlations. The network was estimated using graphical LASSO with extended Bayesian information criterion model selection (γ = 0.5) to obtain a sparse and interpretable structure (Epskamp et al., 2018; Foygel & Drton, 2010). Bridge expected influence (one-step) was computed to identify items linking the two communities (P. J. Jones et al., 2021). Network robustness was assessed using nonparametric and case-dropping bootstrap procedures (1000 iterations). The correlation stability coefficient was calculated to evaluate the stability of edge weights and bridge centrality, with values above 0.25 indicating adequate stability (Epskamp et al., 2018).

## 3. Results

### 3.1. Descriptive Statistics and Correlations

Descriptive statistics and bivariate correlations for all study variables are presented in Table 1. Self-objectification (T1), body shame (T2), and self-disgust (T2) were positively correlated with depression (T3) (all *p*s < 0.001). In contrast, perceived peer connectedness (T1) was negatively correlated with self-objectification, body shame, self-disgust, and depression (all *p*s < 0.001). In addition, body shame and self-disgust were positively associated with depression (all *p*s < 0.001). With respect to demographic variables, gender was significantly associated with self-objectification, body shame, self-disgust, and depression, whereas grade was significantly associated with body shame, depression, and perceived peer connectedness, but not with self-objectification. Supplementary multi-group confirmatory factor analyses were conducted to examine gender measurement invariance for the focal measures. The results showed acceptable configural, metric, and scalar model fit for self-objectification, body shame, self-disgust, perceived peer connectedness, and depressive symptoms. Across the focal scales and invariance models, fit indices were within an acceptable range, with CFI = 0.959–0.980, TLI = 0.957–0.969, RMSEA = 0.047–0.055, and SRMR = 0.023–0.084. These results provided supplementary support for the gender comparability of the focal measures.

### 3.2. Structural Equation Modeling

**Figure 1:**
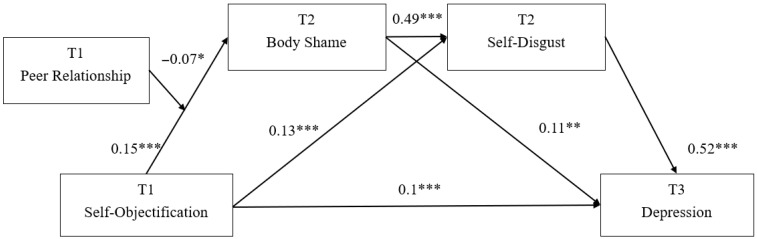
Moderated indirect-effects model linking self-objectification to depressive symptoms. *Note.* *N* = 1181. Path coefficients are standardized beta values. The model controls for gender and grade. Self-objectification was indirectly associated with depressive symptoms through a theoretically specified pathway involving body shame and self-disgust. Perceived peer connectedness moderated the association between self-objectification and body shame. * *p* < 0.05, ** *p* < 0.01, *** *p* < 0.001.

To further examine whether body shame, self-disgust, and perceived peer connectedness were involved in the longitudinal association between self-objectification and depressive symptoms, a moderated indirect-effects model was tested while controlling for gender and grade. Self-objectification at Time 1 was specified as the predictor, body shame and self-disgust at Time 2 as theoretically ordered mediators, perceived peer connectedness at Time 1 as the moderator, and depressive symptoms at Time 3 as the outcome variable. The model demonstrated acceptable fit to the data, χ^2^/df = 5.98, CFI = 0.98, TLI = 0.90, RMSEA = 0.05 (90% CI [0.031, 0.089]), and SRMR = 0.02. The final model with standardized path coefficients is presented in Figure 1. As summarized in Table 2, regarding direct effects, Self-objectification at Time 1 was significantly and positively associated with depressive symptoms at Time 3 (*β* = 0.103, 95% CI [0.054, 0.146], *p* < 0.001). In addition, self-objectification at Time 1 was significantly associated with body shame (*β* = 0.145, 95% CI [0.093, 0.191], *p* < 0.001) and self-disgust at Time 2 (*β* = 0.134, 95% CI [0.076, 0.179], *p* < 0.001). Body shame was significantly associated with self-disgust (*β* = 0.488, 95% CI [0.429, 0.534], *p* < 0.001). With respect to the outcome variable, both body shame (*β* = 0.105, *p* = 0.001) and self-disgust (*β* = 0.520, *p* < 0.001) at Time 2 were significantly associated with depressive symptoms at Time 3.

### 3.3. Moderation Effects

**Figure 2:**
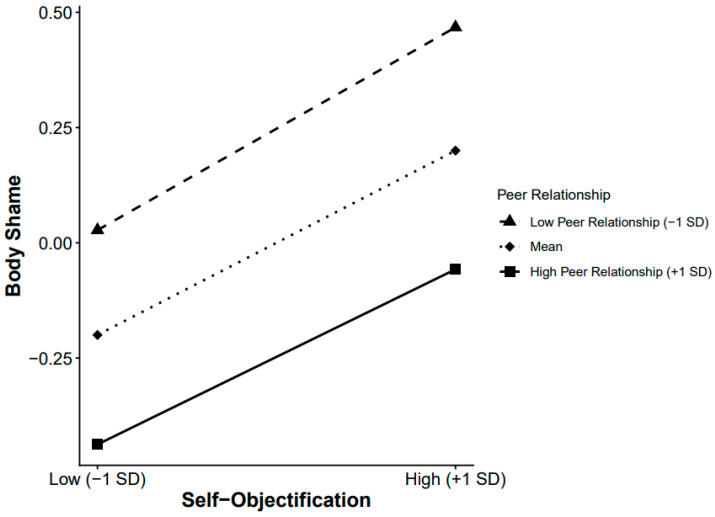
Simple slope plot illustrating the moderating role of perceived peer connectedness in the association between self-objectification and body shame. *Note*. Self-objectification and body shame were standardized. Lines represent the estimated association between self-objectification and body shame at low (−1 SD), mean, and high (+1 SD) levels of perceived peer connectedness. The positive association between self-objectification and body shame was slightly weaker at higher levels of perceived peer connectedness, consistent with a small attenuation pattern.

At the moderation level, perceived peer connectedness was significantly and negatively associated with body shame (*β* = −0.467, *p* < 0.001). The interaction between self-objectification and perceived peer connectedness was also significantly associated with body shame (*β* = −0.072, *p* = 0.029, 95% CI [−0.127, −0.006]), indicating a statistically significant but small moderation effect. To further probe this interaction, simple slope analyses were conducted (see Figure 2). The results showed that self-objectification was significantly and positively associated with body shame at low, mean, and high levels of perceived peer connectedness; however, the strength of this association decreased slightly as perceived peer connectedness increased. Specifically, at low levels of perceived peer connectedness, self-objectification showed a relatively stronger positive association with body shame (*β* = 0.22, *p* < 0.001, 95% CI [0.140, 0.291]). This association was slightly weaker at the mean level of perceived peer connectedness (*β* = 0.20, *p* < 0.001, 95% CI [0.134, 0.279]) and further decreased at high levels (*β* = 0.19, *p* < 0.001, 95% CI [0.126, 0.266]). These findings indicate a statistically significant but small attenuation effect, such that the association between self-objectification and body shame was slightly weaker at higher levels of perceived peer connectedness. Regarding control variables, gender (1 = male and 2 = female) was significantly and positively associated with depressive symptoms (b = 1.090, *p* < 0.001) and body shame (b = 1.301, *p* = 0.002), indicating that girls reported higher levels of these variables than boys. Grade (1 = lower grade and 2 = higher grade) was negatively associated with body shame (b = −1.053, *p* = 0.025) but positively associated with depressive symptoms (b = 0.697, *p* = 0.008).

### 3.4. Mediation and Moderated Mediation Effects

Indirect associations and moderated indirect associations were examined using bias-corrected bootstrap procedures with 5000 resamples. Results indicated a pattern of path-specific moderated indirect associations. The theoretically specified indirect association between self-objectification and depressive symptoms through body shame and self-disgust varied significantly as a function of perceived peer connectedness, as indicated by a significant index of moderated mediation (IMM = −0.003, SE = 0.001, *p* = 0.034, 95% CI [−0.006, −0.001]). Specifically, the conditional indirect associations decreased slightly as perceived peer connectedness increased, with estimates of 0.032, 0.031, and 0.029 at low, mean, and high levels of perceived peer connectedness, respectively; all corresponding 95% confidence intervals excluded zero. In contrast, the indirect association between self-objectification and depressive symptoms through body shame alone was not significantly moderated by perceived peer connectedness (IMM = −0.001, 95% CI [−0.003, 0.001]). In addition, the indirect association involving self-disgust remained significant (β = 0.058, 95% CI [0.037, 0.085]). The total association between self-objectification and depressive symptoms was significant and relatively stable across low, mean, and high levels of perceived peer connectedness (0.191, 0.189, and 0.187, respectively; all *p*s < 0.001). Overall, the model explained 27% of the variance in body shame, 29% in self-disgust, and 39% in depressive symptoms.

### 3.5. Exploratory Item-Level Associations Between Body Shame and Self-Disgust: A Network Analysis

**Figure 3:**
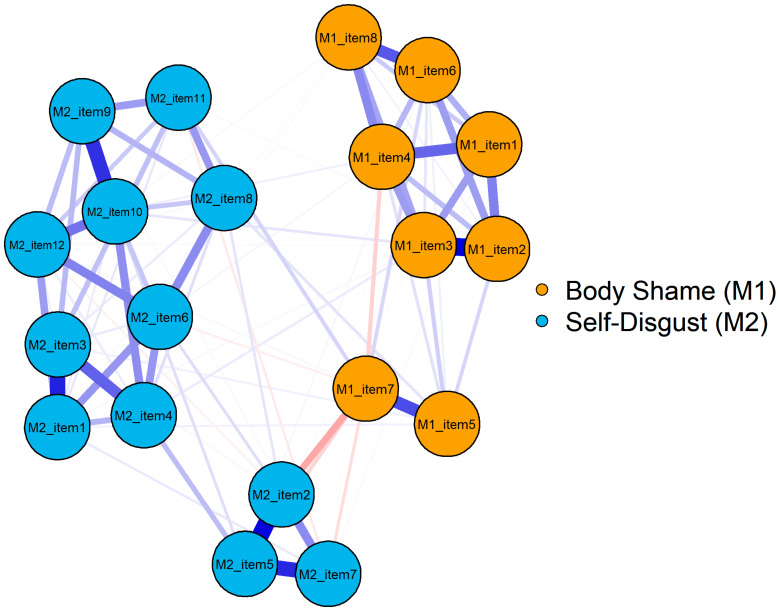
Item-level network structure of body shame and self-disgust. *Note.* Nodes represent individual items of the OBCS body shame subscale (yellow) and the Self-Disgust Scale (blue). Edges represent regularized partial correlations between items, with thicker edges indicating stronger associations. The network was estimated using the EBICglasso algorithm.

To further explore item-level associations between body shame and self-disgust, a regularized partial correlation network was estimated using the EBICglasso algorithm. The network included 8 items of body shame and 12 items of self-disgust. As illustrated in Figure 3, items clustered into two clearly distinguishable and internally cohesive communities corresponding to body shame and self-disgust. Connections between the two communities were predominantly positive, suggesting widespread conditional associations between body shame and self-disgust at the item level.

**Figure 4:**
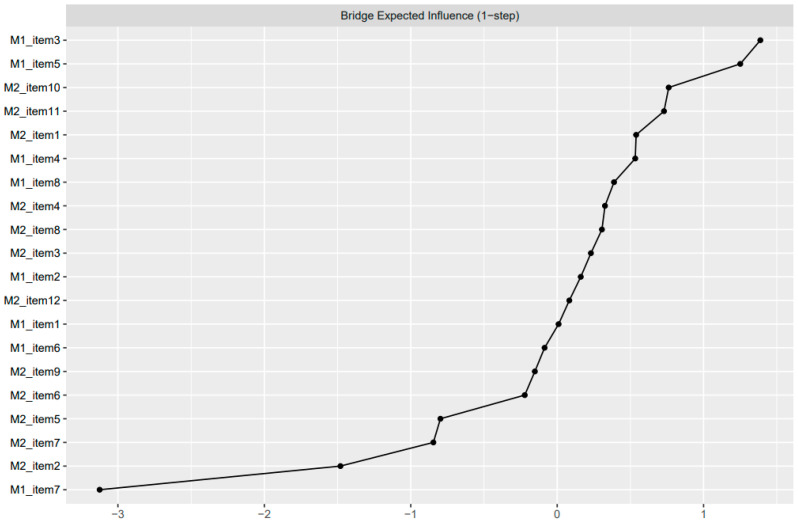
Bridge expected influence of items across body shame and self-disgust communities. *Note.* The figure displays one-step bridge expected influence values for each item, with higher values indicating a greater role in connecting the two symptom communities. M1_item3 (“When I do not look as good as I possibly can, I feel like a bad person”) showed the highest bridge expected influence in the estimated network.

Stability analyses indicated satisfactory precision of edge-weight estimates, and centrality indices demonstrated adequate robustness, supporting the interpretability of the estimated network structure. To identify bridge items linking the two psychological constructs, bridge expected influence (one-step) was computed. As shown in Figure 4, the body shame item M1_item3 (“When I do not look as good as I possibly can, I feel like a bad person”) exhibited the highest bridge expected influence, indicating that it showed the strongest bridge expected influence in connecting the two communities. In addition, M1_item5 within the body shame community, as well as M2_item10 (“I find myself irritating”) and M2_item11 (“I hate certain aspects of my personality”) within the self-disgust community, showed relatively high bridge values, suggesting that these items also contributed to cross-community associations.

Overall, the network findings suggest that the association between body shame and self-disgust can be further characterized at the item level through specific cross-community connections. In particular, M1_item3—linking perceived appearance failure to a global negative evaluation of the self—showed the strongest bridge expected influence. This pattern suggests that appearance-related shame items involving global self-evaluation may represent statistically central points of connection between body shame and self-disgust in the estimated network.

## 4. Discussion

### 4.1. Body Shame and Self-Disgust as Related Forms of Negative Self-Evaluation

The present study found that Time 1 self-objectification was longitudinally associated with Time 3 depressive symptoms through a theoretically specified indirect pathway involving Time 2 body shame and self-disgust. These findings extend objectification theory (Fredrickson & Roberts, 1997) by suggesting that self-disgust, as a broader form of self-directed negativity, may be involved in the longitudinal association between self-objectification and depressive symptoms.

Previous research has identified body shame as the primary emotional consequence of self-objectification and a key mediator linking self-objectification to depression (Grabe et al., 2007; Szymanski & Henning, 2007). However, the current findings suggest that body shame may not be merely an appearance-specific correlate of self-objectification but may also be closely linked to broader negative self-evaluations. Consistent with self-discrepancy theory (Higgins, 1987), adolescents who internalize unrealistic appearance ideals are likely to experience persistent discrepancies between their actual and ideal body selves. During adolescence—a period characterized by heightened social sensitivity and an evolving self-concept—unresolved body-related shame may be associated with broader negative patterns of self-evaluation.

From this perspective, appearance-related discrepancies may be linked to more global negative self-evaluations. In the present study, body shame was strongly associated with self-disgust, which is consistent with the interpretation that appearance-specific shame and broader self-directed negativity are closely connected during adolescence. However, because body shame and self-disgust were assessed at the same wave, the present findings cannot determine whether body shame temporally precedes self-disgust. Unlike body shame, which is more appearance-specific, self-disgust reflects broader rejection of the self as a whole and showed a stronger association with depressive symptoms in the present model (Overton et al., 2008; Spreckelsen et al., 2018).

Overall, these findings suggest that the psychological correlates of self-objectification may extend beyond body-related distress and may involve broader negative self-evaluation, particularly self-disgust as a broader form of self-directed negativity.

### 4.2. Perceived Peer Connectedness as a Modest Contextual Buffer

In addition to the indirect association described above, the present study found that perceived peer connectedness moderated the association between self-objectification and body shame. The positive association between self-objectification and body shame was slightly weaker among adolescents reporting higher perceived peer connectedness.

This finding extends objectification theory by embedding it within a broader socioecological framework. Perceived peer connectedness provides not only emotional support but also diversified sources of social evaluation. High-quality peer interactions are typically accompanied by positive feedback regarding personality, competence, and social roles—domains less centered on appearance (Bukowski et al., 2010; Furman & Rose, 2015; Alsarrani et al., 2022). Such multidimensional validation may reduce the centrality of appearance in adolescents’ self-evaluative systems and may be associated with lower levels of appearance-related shame.

In contrast, adolescents with lower perceived peer connectedness may rely more heavily on appearance-based standards for self-worth. Without diversified validation, appearance may become a more salient criterion for self-evaluation, which may help explain the stronger association between self-objectification and body shame among adolescents with lower perceived peer connectedness (Tiggemann & Slater, 2015). This pattern aligns with the stress-buffering model (Cohen & Wills, 1985), which suggests that social support mitigates the psychological impact of stressors by shaping cognitive and emotional responses.

The moderated indirect association was statistically significant but small, suggesting that perceived peer connectedness may serve as a modest contextual buffer. This pattern indicates that peer connectedness may slightly attenuate the association between self-objectification and body shame, but its practical significance should not be overstated. Thus, perceived peer connectedness is best understood as one contextual resource among multiple individual, family, and sociocultural factors that may shape adolescents’ body-related self-evaluation and emotional adjustment.

### 4.3. Exploratory Item-Level Associations from a Network Perspective

Building upon the macro-level path analyses, the present study employed network analysis to examine micro-level associations between body shame and self-disgust. Unlike traditional latent variable models that treat constructs as homogeneous entities, network analysis conceptualizes psychological phenomena as systems of interacting symptoms, allowing the exploration of bridge nodes linking distinct item communities (Borsboom, 2017).

Results showed that body shame and self-disgust formed two distinct yet interconnected item-level communities. Notably, certain body shame items served as bridges between the two communities. In particular, the item reflecting the tendency to evaluate oneself as a “bad person” when one’s appearance fails to meet expectations exhibited the highest bridge expected influence. This pattern suggests that these items may represent statistically central points of association between appearance-related shame and global self-evaluation within the cross-sectional partial correlation network.

When adolescents equate appearance-based evaluations with global personal worth, body-related shame may be closely connected with broader self-denigrating feelings. These bridge associations may help generate hypotheses for future research on how appearance evaluation is linked with global self-worth. Given that bridge centrality estimates may vary across samples and network specifications, future studies should examine the replicability of these item-level associations in independent longitudinal samples.

### 4.4. Theoretical and Practical Implications, Limitations, and Future Directions

#### Theoretical and Practical Implications

The present study contributes to the literature and practice in several ways. First, using three-wave data, it provides evidence for a theoretically specified longitudinal indirect association linking self-objectification to depressive symptoms via body shame and self-disgust. By incorporating self-disgust as a deeper form of self-directed negative affect, the findings extend objectification theory beyond appearance-specific shame and suggests that appearance-specific negative evaluation and broader self-directed negativity may be closely related in the context of adolescent depressive symptoms. From an applied perspective, this suggests that adolescent mental health support may benefit from addressing not only body-related distress but also broader negative self-evaluative patterns. Second, integrating network analysis with structural equation modeling identified specific bridge items that were strongly associated with both body shame and self-disgust communities. This multi-level approach offers a more fine-grained description of associations between body shame and self-disgust and may help generate hypotheses for future intervention research. Specifically, practitioners may help adolescents distinguish body-related shame from their global sense of self-worth, particularly when adolescents interpret appearance-related concerns as evidence of being a “bad person.” Third, the findings underscore the contextual role of perceived peer connectedness in the association between self-objectification and body shame. These findings suggest that peer connectedness may be considered as one component of school-based social-emotional support programs, while recognizing that its buffering effect was small.

### 4.5. Limitations and Future Directions

Several limitations warrant consideration. First, participants were recruited from a single public secondary school in southwestern China, which limits the generalizability of the findings despite the large sample size and high retention rate. Because the sample consisted of Chinese adolescents from one cultural context, future studies should recruit adolescents from multiple schools, regions, socioeconomic backgrounds, and sociocultural contexts to examine whether the present findings can be replicated in more diverse samples. Second, although the three-wave design provided temporal separation among the predictor, mediators, and outcome, baseline levels of body shame, self-disgust, and depressive symptoms were not available for adjustment. Therefore, the model cannot determine whether the observed associations reflect changes over time rather than stable individual differences, nor can it fully establish the temporal order between body shame and self-disgust because both were assessed at Time 2. Because the focal constructs were not repeatedly assessed at each wave, longitudinal measurement invariance could not be examined. Future studies should repeatedly assess all focal constructs across waves and use autoregressive or cross-lagged models to clarify these temporal processes. Third, only gender and grade were included as covariates. Other potentially important confounders, such as socioeconomic status, BMI, pubertal status, social media exposure, appearance-related feedback environments, and family support, were not available in the present dataset. These factors may be related to adolescents’ body image, perceived peer connectedness, and depressive symptoms, and should be considered in future research. In addition, perceived peer connectedness was indexed using reverse-coded loneliness, which does not fully capture the multidimensional quality of peer relationships. Future studies should include more comprehensive measures of peer support, friendship quality, and peer acceptance, as well as broader individual, family, and sociocultural contextual factors.

## 5. Conclusions

This three-wave study found that self-objectification was longitudinally associated with adolescent depressive symptoms through a theoretically specified indirect pathway involving body shame and self-disgust. The findings suggest that body-related shame and broader self-directed negativity are closely connected in adolescence, highlighting the need for future research to further test their temporal ordering. Perceived peer connectedness modestly attenuated the association between self-objectification and body shame, highlighting a small contextual buffering pattern. The exploratory network analysis further identified specific bridge items linking body shame and self-disgust at the item level. Overall, the results suggest that appearance-based self-evaluation may be associated with later depressive symptoms through broader negative self-evaluative processes. Future research using repeated assessments of all focal constructs, experimental designs, or intervention studies could further clarify the temporal processes underlying these associations.

## Figures and Tables

**Table 1:** Descriptive Statistics and Correlations of Study Variables.

Variable	*M*	*SD*	1	2	3	4	5	6	7
1. Gender	1.52	0.50	1						
2. Grade	1.71	0.45	0.08 **	1					
3. Self-Objectification (X)	29.13	5.69	0.13 ***	0.09 **	1				
4. Body Shame (M1)	25.98	4.81	0.20 ***	0.13 ***	0.22 ***	1			
5. Self-Disgust (M2)	37.27	5.94	0.19 ***	0.15 ***	0.24 ***	0.52 ***	1		
6. Depression (Y)	10.54	3.13	0.26 ***	0.17 ***	0.26 ***	0.28 ***	0.59 ***	1	
7. Perceived peer connectedness (W)	12.18	4.77	−0.15 ***	−0.13 ***	−0.15 ***	−0.48 ***	−0.57 ***	−0.41 ***	1

*Note. N* = 1181. Gender was coded as 1 = male and 2 = female; grade was coded as 1 = lower grade and 2 = higher grade. *M* and *SD* represent the total scores of each scale. Diagonal elements are 1.00, and off-diagonal elements are Pearson correlation coefficients. ** *p* < 0.01, *** *p* < 0.001.

**Table 2:** Regression Results for the Moderated Indirect-Effects Model Linking Self-Objectification to Depressive Symptoms.

Outcome Variables	Predictive Variables	*R* ^2^	*SE*	*β*	*t*
Body shame T2	Self-objectification T1	0.27	0.040	0.145	5.28 ***
	Perceived peer connectedness T1		0.031	−0.467	−15.07 ***
	SO × Perceived peer connectedness		0.033	−0.072	2.19 *
	Gender		0.026	0.080	3.09 **
	Grade		0.026	−0.058	−2.26 *
Self-disgust T2	Self-objectification T1	0.29	0.028	0.134	4.72 ***
	Body shame T2		0.029	0.488	16.76 ***
	Gender		0.024	−0.051	−2.10 *
	Grade		0.025	−0.008	−0.31
Depression T3	Self-objectification T1	0.39	0.025	0.103	4.05 ***
	Body shame T2		0.031	0.105	3.34 **
	Self-disgust T2		0.028	0.520	18.71 ***
	Gender		0.023	0.114	4.99 ***
	Grade		0.025	0.066	2.66 **

*Note*. *N* = 1181. *β* values are standardized regression coefficients. SE values refer to the standard errors of the corresponding unstandardized regression coefficients. The interaction term represents the product of self-objectification and perceived peer connectedness. R^2^ values indicate the proportion of variance explained in each outcome variable. Perceived peer connectedness was indexed by reverse-coded loneliness scores, with higher scores indicating lower loneliness and greater perceived connectedness. * *p* < 0.05, ** *p* < 0.01, *** *p* < 0.001.

## Data Availability

The data underlying the findings of this study can be obtained from the corresponding author upon a reasonable request.
